# A novel neural-evolutionary framework for predicting weight on the bit in drilling operations

**DOI:** 10.1038/s41598-023-45760-6

**Published:** 2023-10-28

**Authors:** Masrour Dowlatabadi, Saeed Azizi, Mohsen Dehbashi, Hamed Sadeqi

**Affiliations:** 1 Department of Electrical Engineering, Science and Research Branch, Islamic Azad University, Tehran, Iran; 2 Chemical Engineering, Oklahoma State University, Stillwater, OK USA; 3 Institute of Physics, Center for Science and Education, Silesian University of Technology, Konarskiego 22B, 44-100 Gliwice, Poland; 4 Information Technology Department, Informatic University of Applied Sciences, Tehran, Iran

**Keywords:** Engineering, Geology, Petrology

## Abstract

This study compares the performance of artificial neural networks (ANN) trained by grey wolf optimization (GWO), biogeography-based optimization (BBO), and Levenberg–Marquardt (LM) to estimate the weight on bit (WOB). To this end, a dataset consisting of drilling depth, drill string rotational speed, rate of penetration, and volumetric flow rate as input variables and the WOB as a response is used to develop and validate the intelligent tools. The relevance test is applied to sort the strength of WOB dependency on the considered features. It was observed that the WOB has the highest linear correlation with the drilling depth and drill string rotational speed. After dividing the databank into the training and testing (4:1) parts, the proposed LM-ANN, GWO-ANN, and BBO-ANN ensembles are constructed. A sensitivity analysis is then carried out to find the most powerful structure of the models. Each model performs to reveal the relationship between the WOB and the mentioned independent factors. The performance of the models is finally evaluated by mean square error (MSE) and mean absolute error criteria. The results showed that both GWO and BBO algorithms effectively help the ANN to achieve a more accurate prediction of the WOB. Accordingly, the training MSEs decreased by 14.62% and 24.90%, respectively, by applying the GWO and BBO evolutionary algorithms. Meanwhile, these values were obtained as around 9.86% and 9.41% for the prediction error of the ANN in the testing phase. It was also deduced that the BBO performs more efficiently than the other technique. The effect of input variables dimension on the accuracy and training time of the BBO-ANN clarified that the most accurate WOB predictions are achieved when the model constructs with all four input variables instead of utilizing either three or two of them with the highest linear correlation. It was also observed that the training stage of the BBO-ANN model with four input variables needs a little more computational time than its training with either two or three variables. Finally, the accuracy of the BBO-ANN model for the WOB prediction has been compared with the multiple linear regression, support vector regression, adaptive neuro-fuzzy inference systems, and group method of data handling. The statistical accuracy analysis confirmed that the BBO-ANN is more accurate than the other checked techniques.

## Introduction

The choice to use drilling processes in the upstream petroleum sector instead of more expensive activities is a crucial decision. This strategic shift holds significant importance and could lead to a substantial reduction in operational costs^1,2^. There are different parameters in the drilling rig operation (i.e., technical, economic, and human parameters) that have a high influence in this regard^3^. In the drilling industry, the relationship between drilled depth and time can be highly changed using different parameters. This relationship arises from the rate of penetration in the rock^2,4^. In the case of the drilling program, the meticulous selection of parameters that influence the rate of penetration into the formation is crucial and can potentially augment the penetration rate of the drill into the rock. The higher drilling rate can be due to the higher rate of penetration, which can decrease drilling time and finally decrease the total operation cost (e.g., Improving and predicting the impressive parameters can enhance the penetration rate for a specific zone until around 50 m that drilling rate is around 37 m per day. Since the wells of Iran are classified as deep wells, improvement and prediction of penetration rates in drilling wells are very significant^5^.

The most appropriate penetration rate within the formation can be obtained by utilization of calculating approaches that can increase the drilling rate and may lead to lower costs by comparing and studying the impressive parameters by taking into account the mud utilized and the drill bit and hydraulics type as well as the rig power^6^. The least error can be achieved by considering impressive factors of the volume flow rate of drilling fluid, the formation rate of penetration, density of drilling fluid drill string rotational speed (RPM), the pressure of standpipe, size/type of drill bit along with kind of formation the relation among the weight on the drill bit and the drilling factors.

The above-mentioned parameters consist of the following factors:

1. It is known that the higher used weight does not presently mean a higher drilling rate. Weight on a bit is the weight exerted on the drill bit that is named weight on bit (WOB). The most important factor of the drilling rate is when the driller attempts to identically use the optimized weight of the drill string for the bit under the well bottom (e.g., in some sticky substrates, the higher weight leads to the bit falling within the layer. It can barricade the dig of the formation and the drill string rotation, and sometimes causes the drill string traps in the form or even cutting the drill string.
2. The rate of Penetration (ROP) into the formation shows the drilling rate. In this way, the drill adjustment attempts to retain it at the highest feasible level to the lowest cost along with the lowest risk of the operation. ROP in an operation team is commonly called and introduced as the per meter depth drilled (with the unit of time).
3. The drilling depth (Depth) along with the type and hardness of the formation have been classified as the factors that affect the size and type of bit (Rock Bit or PDC Bit).
4. The volumetric flow rate of drilling fluid (GPM) is used with the driller based on drilling states. It includes the size of the drill bit as well as the diameter of the driller nozzles, depth, and pressure of the standpipe. Researchers are trying to retain it at a constant amount.
5. The density of drilling fluid has been considered as one of the factors that is commonly used when hydrostatic pressure in the well depth is more than the formation pressure that prohibits the fluid formation flow of the well. It is commonly attempted to identify and use the optimized fluid density during drilling and determining the optimized fluid density while taking into account the formation layer and well situations.
6. The density of drilling fluid is calculated as the unit of pound per cubic foot. In addition, for drilling operations, the fluid density is named mud weight (i.e., MW).
7. Considering drilling conditions such as depth and formation type, one of the crucial parameters that is sought to be utilized optimally is the rotational speed of the drill string, measured in rounds per minute (rpm).
8. The formation type that is being drilled varies in the case of geology. This leads to the enhancement of the drilling depth. The layers of formation regulation are usually similar in distinct points of a particular oilfield and even sometimes at the same depths, various wells commonly have the same and unchanged structures.
9. Another factor that affects the drilling rate and states is the fluid pressure pumped in the drill string. The drilling fluid can be pumped by taking into account the reservoirs in the drilling string. The drilling fluid, upon exiting the drill bit, should return to its initial level in the reservoirs, both in the annular space surrounding the drill string and within the well. The fluid pressure exerted by the drill depends on several factors, such as the density of the drilling fluid, the effectiveness of the pumps, the pressure drop across the drill nozzles, the pressure drop within the drill string, and the volumetric flow rate determined by the driller.

Many scholars are concerned about the optimization and simulation of the procedure utilizing the common mathematical methods due to the high variation of the parameters involved^7–11^. It is important to note that simulating systems by usual mathematical patterns like differential equations for complex systems that contain uncertainties is known as a relatively good method without high effective performance. Hence, many studies have used artificial intelligence and deep learning models to approximate key variable of different processes, including the drilling operations^12–16^.

Furthermore, some drawbacks of typical intelligent models have driven scholars to employ hybrid evolutionary algorithms to achieve a better approximation of the desired variable^17^. In this sense, Anemangely and Ramezanzadeh optimized the performance of an artificial neural network (ANN) by using a cuckoo optimization algorithm (COA) and particle swarm optimization algorithm for predicting the drilling rate^18^. They concluded that the combination of the neural network with COA could achieve high accuracy in the mentioned utilization. Moraveji and Naderi successfully employed a bat algorithm for identifying the optimal range of parameters for maximizing the drilling rate of penetration^8^.

This research applies machine learning techniques to predict the WOB as a function of different combinations of depth, RPM, ROP, and GPM as the input variables. Firstly, the relevance test sorted the impact of each independent variable on the WOB. Then, two wise optimization algorithms, namely grey wolf optimization (GWO) and biogeography-based optimization (BBO), are applied to improve the prediction capability of artificial neural networks in estimating the WOB. Although ANNs have been promisingly used, the authors came across a few studies that conduct the optimization of ANNs for prevailing its computational drawbacks in the mentioned field. Therefore, two novel ensembles of GWO-ANN and BBO-ANN are designed, and their best structure is determined by executing a sensitivity analysis. The effect of input variables dimension on the accuracy and training time of the best neural-evolutionary framework has also been investigated in this study. The prediction accuracy of the best neural-evolutionary framework is compared with the traditional ANN, multiple linear regression (MLR), support vector regression (SVR), adaptive neuro-fuzzy inference systems (ANFIS), group method of data handling (GMDH).

## Data preparation and statistics

2250 data samples, comprising four independent variables (i.e., drilling depth, RPM, ROP, and GPM), as well as WOB as the dependent variable, are used to develop the intelligent models of this study. The key statistical parameters related to this databank are summarized in Table 1.

**Table 1:** Statistical analyses of the WOB and effective parameters.

Features	Variable	Descriptive index					
		Mean	Median	SD	Min	Max	Count
Depth (m)	Input	2559.33	2307.90	426.07	2149.80	3591.00	2250
RPM (1/min)	Input	105.81	75.85	46.50	50.00	192.42	2250
ROP (m/hr)	Input	5.16	4.62	3.52	0.22	43.42	2250
GPM (gal/min)	Input	465.75	473.10	95.75	250.22	915.59	2250
WOB (Klb)	Output	11.11	9.75	5.93	1.11	31.91	2250

**Figure 1:**
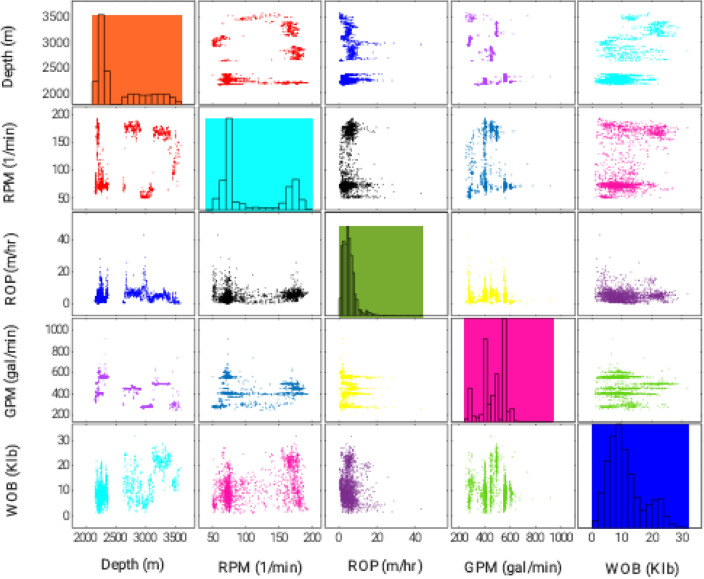
Pair plot of the involved variables in the present study.

The histogram of all independent and dependent variables as well as their scatterplots (known as the pair plot) is presented in Fig. 1.

1800 samples (80% of the whole dataset) are used to perform the learning phase of the proposed models. Indeed, this learning phase helps the intelligent tools to understand the relationship between the WOB and its influential factors. Then, the capability of the developed networks is evaluated by the remaining 450 samples (20% of the whole dataset), which were not seen by the trained models in the learning phase. This stage, which is known as the testing phase, monitors the generalization ability of the trained models against some unseen data samples.

### Feature importance analysis

**Figure 2:**
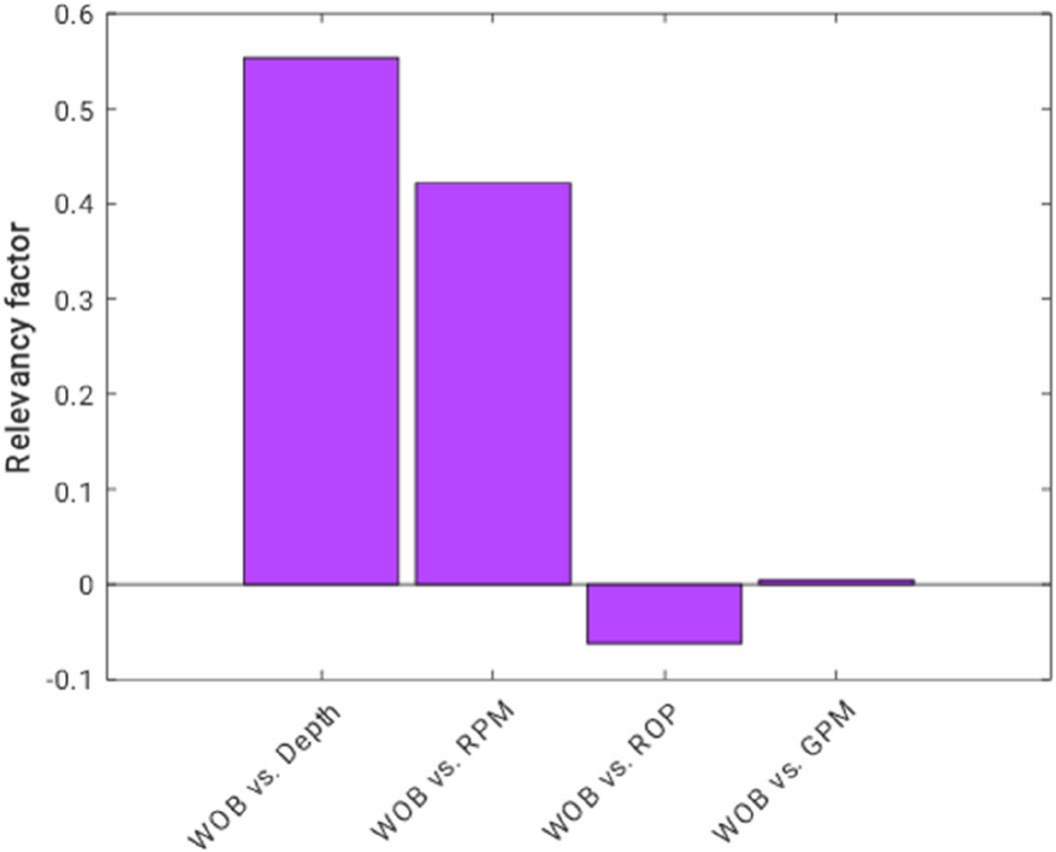
Results of the feature importance analysis.

Feature importance analysis is a very important step before the development of predictive models to estimate the target WOB. This analysis helps understand the level of WOB sensitivity on independent factors. The results of applying Pearson method^19^ on the available databank to reveal the importance level of features have been depicted in Fig. 2. This figure clarifies that the Depth and RPM are the most important features for determining the target, i.e., WOB.

The p-values between WOB and each independent variable are reported in Table 2. This analysis claimed that depth and RPM are highly significant statistically. In addition, ROP and GPM have been identified as very significant and not significant variables, respectively.

**Table 2:** The observed p-values between WOB and the input variables.

Independent variable	p-values	Interpretation^20^
Depth	< 0.0001	Highly significant
RPM	< 0.0001	Highly significant
ROP	0.003	Very significant
GPM	0.833	Not significant

## Methodology

After providing a proper dataset, it is divided into two parts for training and testing the proposed ANN, GWO-ANN, and BBO-ANN networks. To develop the mentioned ensembles, the GWO and BBO optimization techniques are coupled with the ANN. Then, the best structures are used to estimate the WOB. The results are evaluated utilizing two statistical criteria, i.e., mean square error (MSE) and mean absolute error (MAE). Equations (1) and (2) formulate the MSE and MAE. Finally, the results of the ensembles are compared with the typical ANN to examine the effect of the applied metaheuristic algorithms. Moreover, the predictive mathematical equation of the most capable network is presented^21^.

1 $$MSE = \left( {1/N} \right)\sum\limits_{i = 1}^{N} {\left( {WOB_{i}^{act} - WOB_{i}^{cal} } \right)^{2} }$$

2 $$MAE = \left( {1/N} \right)\sum\limits_{i = 1}^{N} {\left| {WOB_{i}^{act} - WOB_{i}^{cal} } \right|}$$

in which the superscripts of *act* and *cal* denote the actual and calculated values of WOB, respectively. The term *N* represents the number of instances.

### Artificial neural network

**Figure 3:**
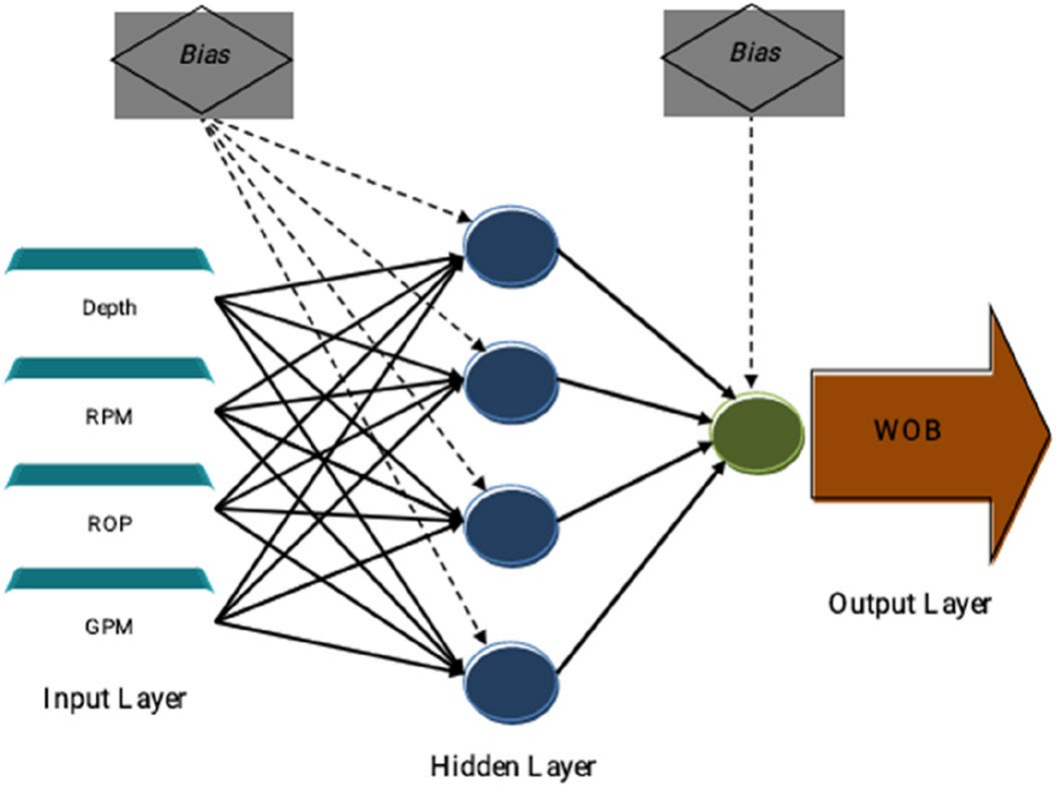
The typical MLP structure.

The ANN algorithm can be used to solve different non-linear engineering problems^22^. McCulloch and Pitts^23^ suggested the idea of this algorithm, which mimics the biological neural procedures. As other machine learning methods, two different groups of data are needed to develop the ANN. The first one includes the most data named training dataset that is employed for pattern recognition via developing mathematical relations. The second group analyzes the performance of the developed network named testing database. Multi-layer perceptron (MLP) is known as one of the most appropriate methods between various types of ANNs that were employed in many studies^24^. In the present paper, we have used the algorithm of LM (Levenberg–Marquardt)^25^, GWO, and BBO to accomplish the training phase of MLP neural networks. A typical MLP structure related to our study is shown in Fig. 3.

After performing the training method in the process of BP, the efficiency error is calculated based on the difference between the exact and estimated response variable. For updating the computational weights (*W*) as well as biases (*b*), the efficiency error is propagated backward. We consider *Out*, *T* = {${Depth}_{i}$, ${RPM}_{i}$, ${ROP}_{i}$, ${GPM}_{i}$; i = 1, 2, …, N}, and *F* as output vectors, input matrix, and the activation function of the MLP model. After that, the jth computational unit performance (i.e., the neurons of the MLP) can be described as follows:

3 $$Out_{j} = F \, \left( {\sum\limits_{m = 1}^{M} {T_{m} W_{mj} + b_{j} } } \right)$$

It should be mentioned that the current study uses the tangent sigmoid (tansig) and linear activation functions in the hidden and output layers, respectively^26^.

### Grey wolf optimization

**Figure 4:**
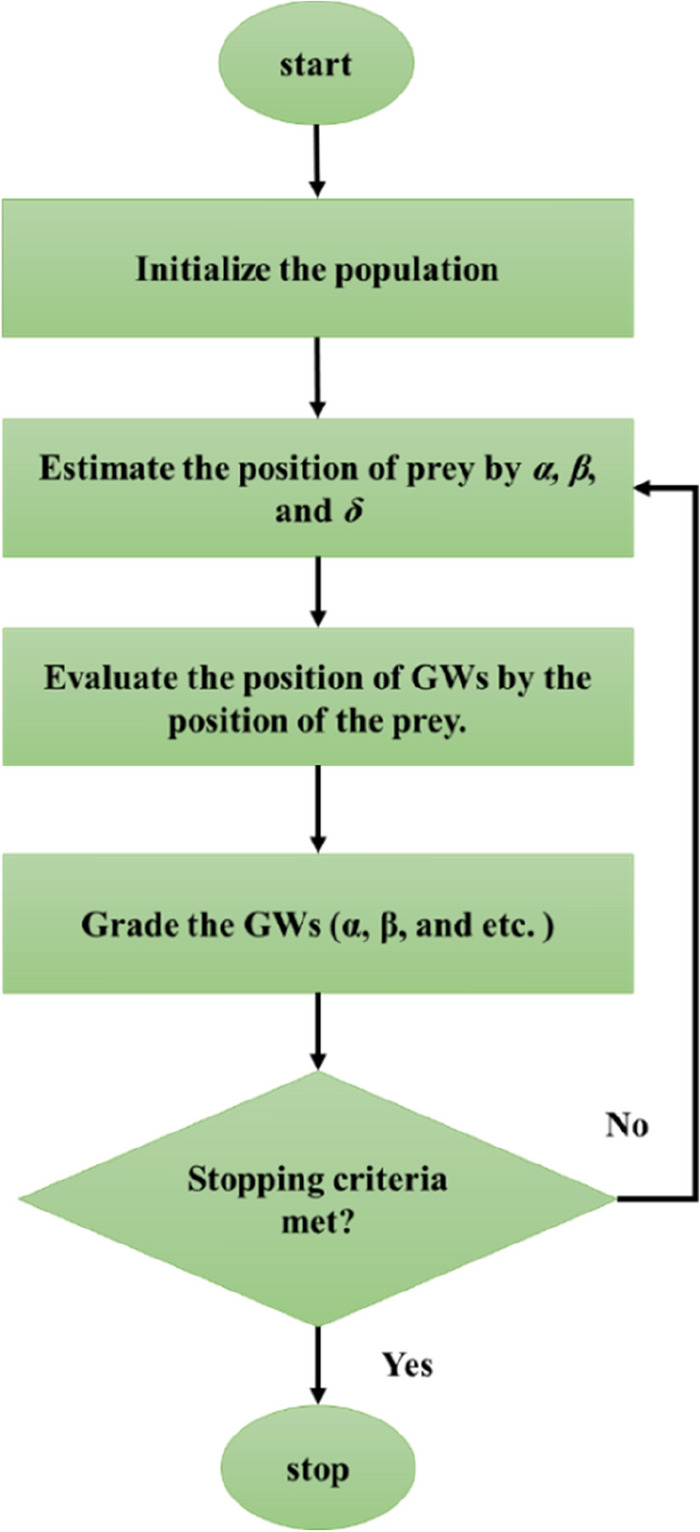
The working procedure of the GWO algorithm.

The grey wolf optimization has been suggested as a progressive evolutionary algorithm^27^. GWO follows the behavior of grey wolf herds, which is a type of social hierarchy. For the first time, this algorithm is suggested by Mirjalili et al.^28^. A flowchart of this algorithm is shown in Fig. 4. A group of wolves in the wolf flock (named alpha, *α*) is the head of the herd involving several male and female wolves that are decision-makers flocks for hunting and searching. A group of wolves in the wolf flock (named beta, *β*) assist and obey the first group of decision-makers. The Beta wolves establish the discipline of the herd, and this is their main duty. When alpha (*α*) wolves retire or die, beta (*β*) wolves can substitute instead of them. The next group of wolves that act as scouts, sentinels, hunters, etc., and have the weakest relationships and babysitters are named delta (*δ*) (omega (*ω*) wolves). Omega (*ω*) wolves are the weakest wolves in the flock, and it is likely to see internal fights without omega (*ω*) wolves. These wolves can participate in hunting, and this is their main social behavior with a social hierarchy^29,30^.

Three base stages of the algorithm of GWO are (a) approaching the target, following, detecting, and (b) circumambienting the target, as well as (c) attacking the prey^31^. Alpha (*α*) is used as the highest-fitted solution, and after that *β*, *δ*, and *ω*. The encompassing formula is obtained as below:

4 $$\overrightarrow {D} = \left| { \, \overrightarrow {S} \cdot \, \overrightarrow {P}_{p} (t) - \overline{P} (t) \, } \right|$$

5 $$\overrightarrow {P} (t + 1) = \overrightarrow {P}_{p} (t) - \overrightarrow {F} \, \cdot \, \overrightarrow {G}$$

where t stands for the time of iteration. A new position for a wolf is proposed by the vector of $\overrightarrow{D}$. The $\overrightarrow{F}$ and $\overrightarrow{S}$ stand for the coefficient vectors, and $\overrightarrow{{P}_{p}}$ and $\overrightarrow{P}$ show the prey and wolf positions, respectively.

We assume that *δ, α,* and* β* are more trustworthy knowledge around the prey place, the most appropriate solutions for considered positions might be updated. After that, other wolves can then register the positions.

6 $$\overrightarrow {D}_{\alpha } = \left| { \, \overrightarrow {S}_{1} \, \cdot \overrightarrow {P}_{\alpha } - \overrightarrow {P} \, } \right|$$

7 $$\overrightarrow {D}_{\beta } = \left| { \, \overrightarrow {S}_{2} \cdot \, \overrightarrow {P}_{\beta } - \overrightarrow {P} \, } \right|$$

8 $$\overrightarrow {D}_{\delta } = \left| { \, \overrightarrow {S}_{3} \cdot \, \overrightarrow {P}_{\delta } - \overrightarrow {P} \, } \right|$$

9 $$\overrightarrow {P}_{1} = \overrightarrow {P}_{\alpha } - F_{1} \cdot \overrightarrow {D}_{\alpha }$$

10 $$\overrightarrow {P}_{2} = \overrightarrow {P}_{\beta } - F_{2} \cdot \overrightarrow {D}_{\beta }$$

11 $$\overrightarrow {P}_{3} = \overrightarrow {P}_{\delta } - F_{3} \cdot \, \overrightarrow {D}_{\delta }$$

12 $$\overrightarrow {P} (t + 1) = \frac{{\overrightarrow {P}_{1} + \overrightarrow {P}_{2} + \overrightarrow {P}_{3} }}{3}$$

Finally, in this method, when a target stands the attack is accomplished. Note that the *F* includes random variables among − 2*α* and 2*α*. This shows that the wolves attack the target ($\left|A\right|$ < 1) or look for a better one ($\left|A\right|$ > 1)^29^.

### Biogeography-based optimization

**Figure 5:**
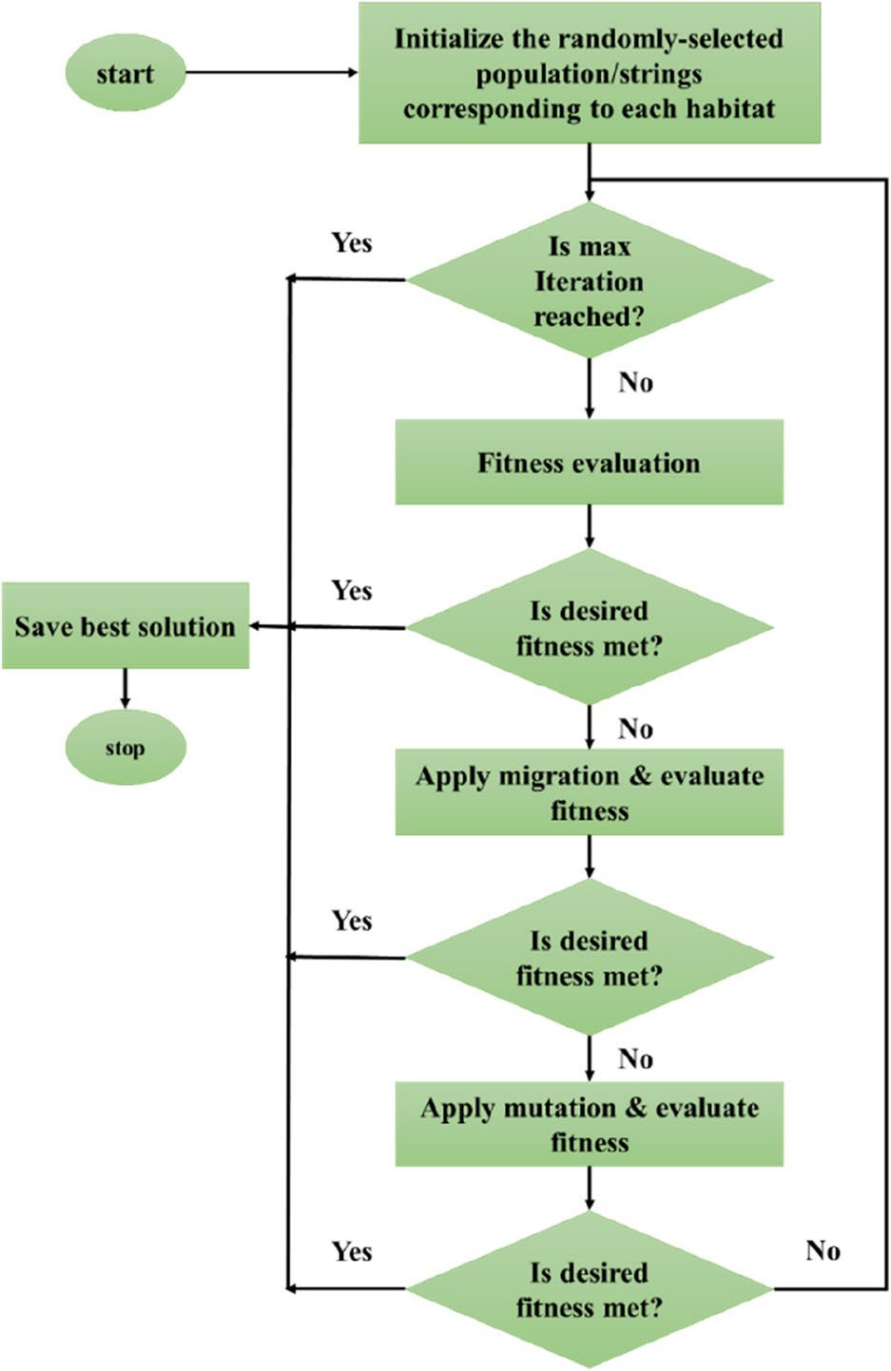
The operation procedure of the BBO algorithm.

Simon^32^ introduced biogeography-based optimization that comes from the biogeography knowledge and dispensation of various species. This model has been classified as a population-based search method, and Mirjalili et al.^33^ applied this algorithm to MLP to optimize its efficiency. Figure 5 denotes the flowchart of the BBO. This model begins using the generation of a so-called random population called “habitat.” These parameters show possible solutions that have been obtained from the habitat suitability index (HSI). Moreover, the suitability index variable (SIV) can be used to evaluate the habitability of the habitats and zones. More precisely, an SIV shows the population of the candidate solutions and is classified as a group of real numbers. The BBO extracts two distinct practices (i.e., migration and mutation). The basic purpose is to improve the quality of the possible solutions by enhancing them according to other existing solutions. In this stage, λ_g_, an immigration rate, is considered for deciding about the need for correction of each SIV^34^. In addition, emigration rates (μ_g_) can be used to choose the solution that migrates. It should be noted that other metaheuristic methods are retained constant away from this act to pass probabilistic corruption^35^.

Abrupt variations in the case of HSI values can be due to some normal hazards threatening geographical zones. The habitat likely strays due to equilibrium HIS. This method is called mutation, and the probability of each species count determines its rate. This probability is expressed as below:

13 $$P_{g}^{f} = \left\{ {\begin{array}{*{20}l} { - (\lambda_{g} + \mu_{g} )P_{g} + \mu_{g + 1} P_{g + 1} } \hfill &\quad {S = 0,} \hfill \\ { - (\lambda_{g} + \mu_{g} )P_{g} + \lambda_{g - 1} P_{g - 1} + \mu_{g + 1} P_{g + 1} } \hfill &\quad {1 \le S \le S_{\max } - 1,} \hfill \\ { - (\lambda_{g} + \mu_{g} )P_{g} + \lambda_{g - 1} P_{g - 1} } \hfill &\quad {S = S_{\max } .} \hfill \\ \end{array} } \right.$$

in which *S* stands for the species number for a region.

To decide whether the relation will change or not, any population relation becomes a possibility. This additionally shows whether the relation is a solution to the existing issue or not. The proximity of the solution to the final solution can be obtained by probability value. A higher probability value means that the solution is near to the overall solution^36^.

### Stopping criteria

The involved optimization algorithms follow an iterative procedure to minimize the deviation between actual and predicted values of the WOB in the learning phase. The weights and biases of the ANN model are continuously updated by the backpropagation method to reduce this deviation as much as possible. Reaching the maximum number of iterations, converging to a prespecified accuracy, and experiencing a minimum error gradient are the most well-known criteria for stopping the learning phase.

## Results and discussion

As explained, this study applies two novel optimization algorithms to perform the learning stage of artificial neural networks for estimating the WOB. To this end, after providing the required data, utilizing the programming language of Matlab (Version, 2019b), the GWO and BBO metaheuristic algorithms are coupled with an MLP neural network to find the best computational parameters of this tool. More specifically, the mathematical equation of the MLP is introduced as the main problem with the weights and biases as the variables. The mentioned algorithms aim to find the most appropriate values of the weights and biases, which produce the most consistent responses. Then, the optimized ANN is reconstructed by applying the new parameters.

### Finding the optimal structure of intelligent models

**Figure 6:**
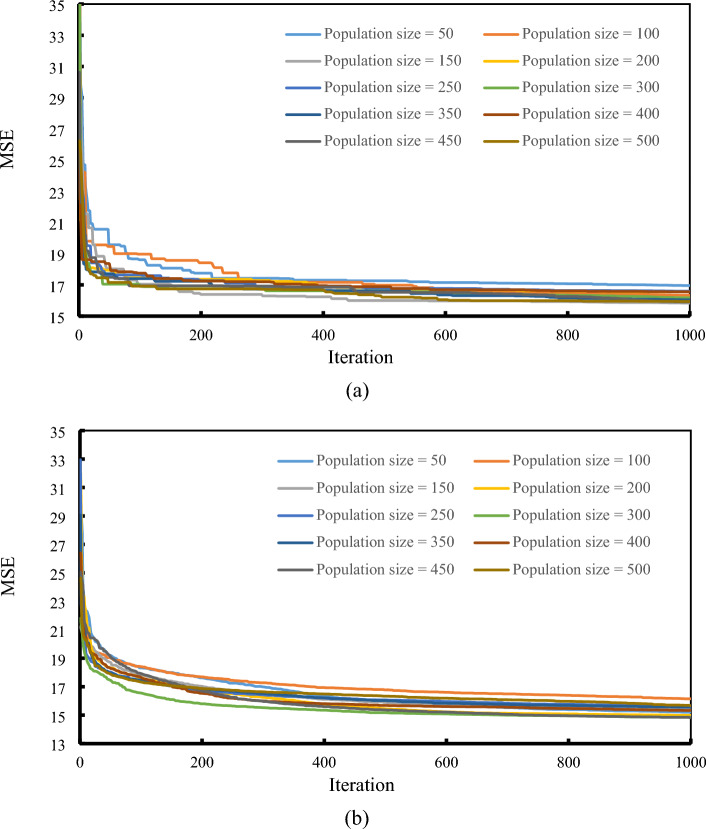
Sensitivity analysis for the (**a**) GWO-ANN and (**b**) BBO-ANN.

It is well understood that diverse structures of intelligent models yield distinct results. Therefore, in order to pinpoint the most dependable configurations for the proposed GWO-ANN and BBO-ANN ensembles, a comprehensive process of trial and error was conducted. As explained supra, both GWO and BBO are population-based techniques. Ten different structures, i.e., with ten population sizes varying from 50 to 500 with 50 intervals, were designed and tested within 1000 repetitions. Remarkably, the MSE error criterion was defined as the cost function (CF) to evaluate the accuracy of the models. Over time, the individuals of the optimization algorithms aim to find a more fitted solution, which results in a decrease in error. It is worth noting that to evaluate the repeatability of the used models, each network was performed five times. The convergence curves of the implemented GWO-ANN and BBO-ANN are presented in Fig. 6a,b, respectively.

As the charts illustrate, both GWO and BBO reduced the majority of errors in the first 300 iterations. Comparing the accuracies, it was revealed that the lowest MSE was obtained for the GWO-ANN and BBO-ANN with population sizes of 150 and 450, respectively. However, other population sizes presented a close MSE. Taking into account the computational time, the elite GWO-ANN took about 37.13 min to find the most proper structure of the ANN. This time was obtained about 107.62 min for the best BBO-ANN network.

### Model assessment

**Figure 7:**
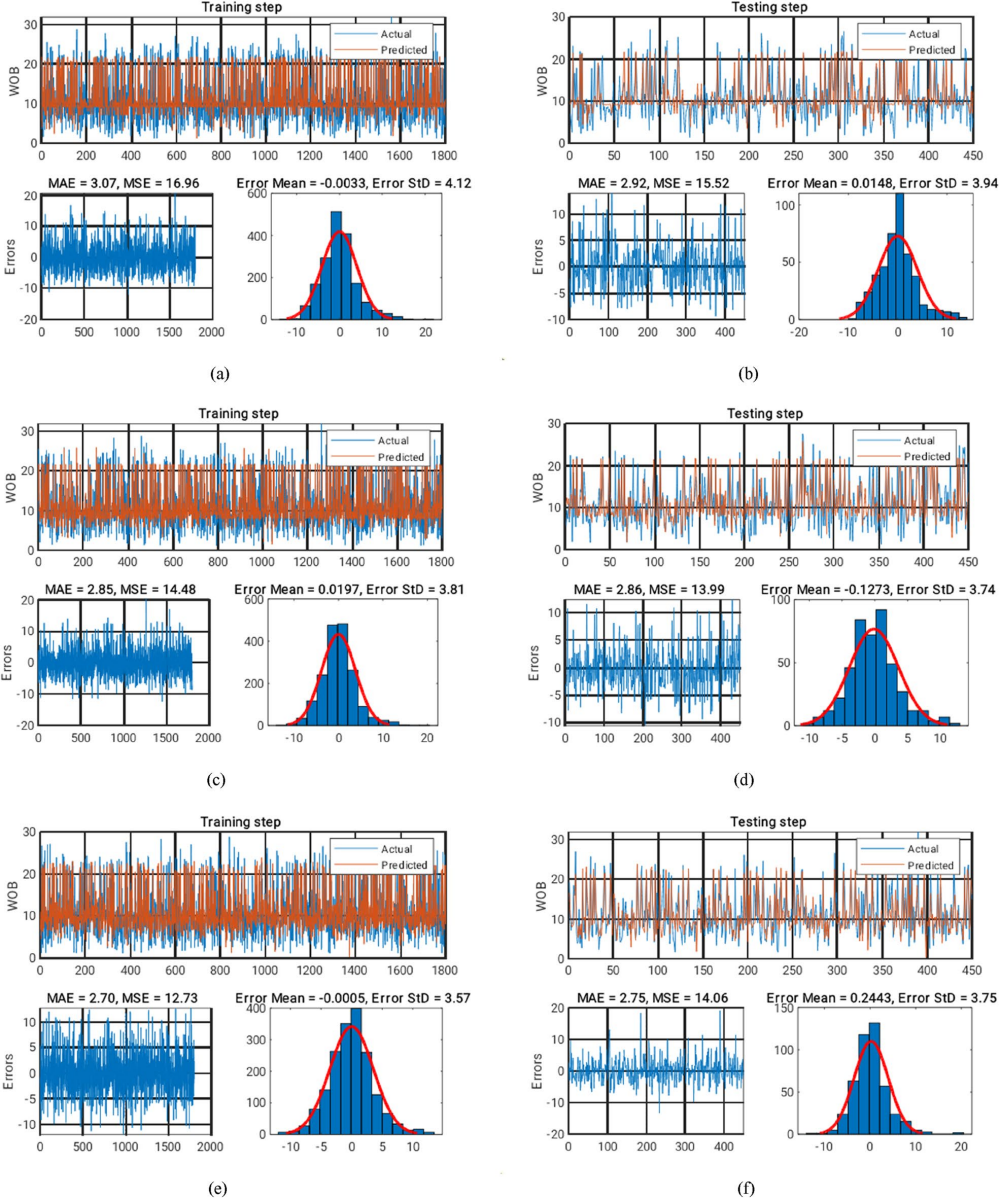
The results obtained for the LM-ANN model in (**a**) training and (**b**) testing stages. The results obtained for the GWO-ANN model in (**c**) training and (**d**) testing stages. The results obtained for the BBO-ANN model in (**e**) training and (**f**) testing stages.

Then, the MSE and MAE of the prediction responses of the implemented LM-ANN, GWO-ANN, and BBO-ANN in the training and testing stages were calculated to compare their efficiency. The results are shown in Fig. 7a–f. In this figure, the graphical comparison between the actual and predicted WOBs is presented, as well as the calculated error (i.e., indicating the difference between the targets and outputs) and the histogram of the errors.

As is seen, all three models performed satisfactorily in both recognizing and predicting the pattern of the WOB influenced by depth, RPM, ROP, and GPM. Moreover, the calculated MSEs indicate that the GWO and BBO algorithms reduced the learning error of the LM-ANN by 14.62% and 24.90% (from 16.96 to 14.48 and 12.73), respectively. Also, the obtained MAEs attest to these results with 7.70% and 10% (from 3.07 to 2.85 and 2.70) decrease in the mean absolute value of the ANN training error, respectively, by applying the GWO and BBO algorithms.

**Figure 8:**
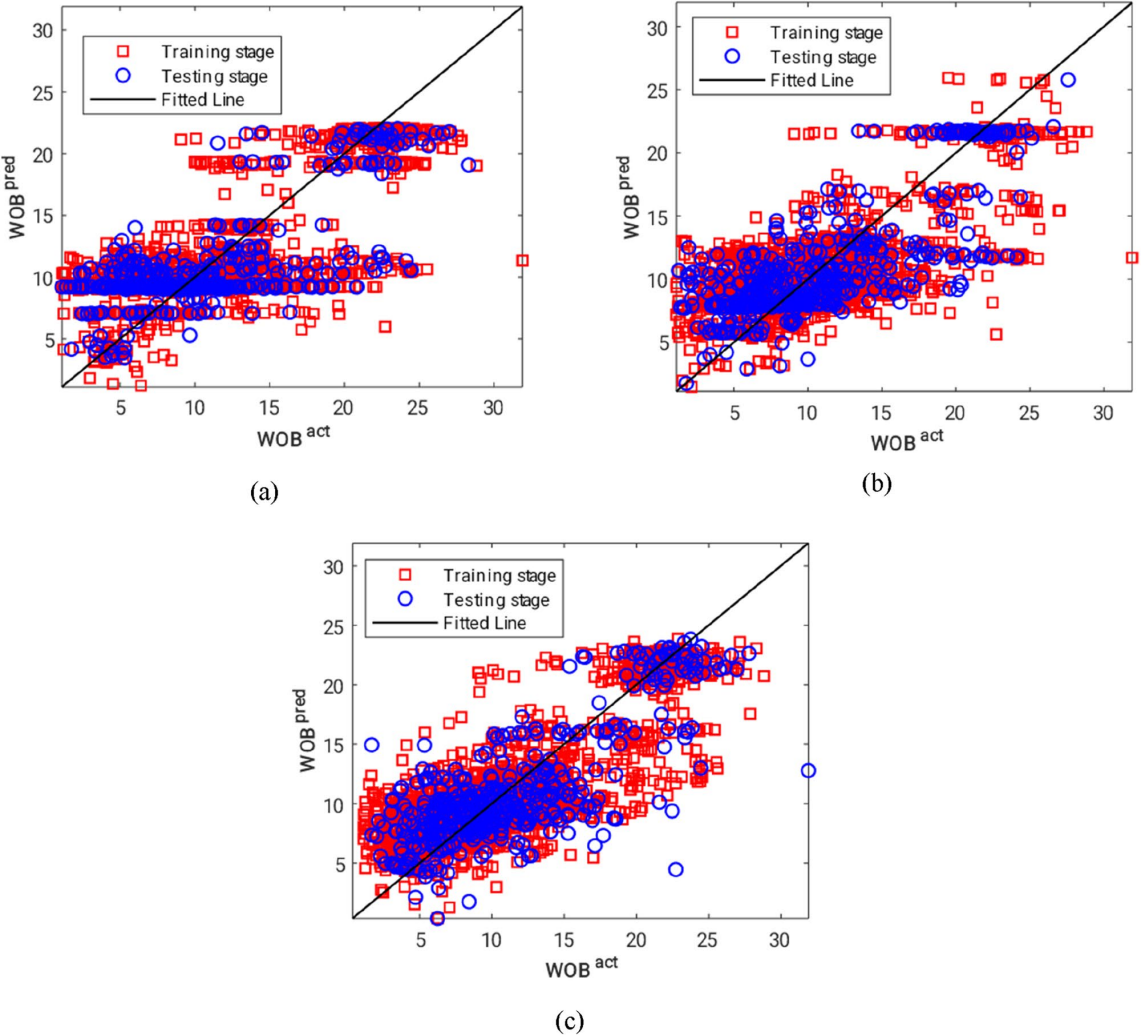
The cross-plots of (**a**) LM-ANN, (**b**) GWO-ANN and (**c**) BBO-ANN models.

As for the testing phase, it was revealed that the reinforced ANN enjoys more prediction capability (in comparison with the typical one), regarding the observed decrease in the calculated MSEs and MAEs. Accordingly, the testing MSE declined by 9.86% and 9.41% (from 15.52 to 13.99 and 14.06). Additionally, these values were obtained as 2.05% and 5.82% (from 2.92 to 2.86 and 2.75) for the testing MAEs. Furthermore, the cross-plots associated with the estimated WOBs by the LM-ANN, GWO-ANN, and BBO-ANN are presented in Fig. 8a–c, respectively.

From a comparison point of view, it is deduced that the BBO surpassed the GWO in optimizing the ANN. In other words, the solution provided by the BBO algorithm for solving the mathematical equation of the ANN developed (for estimating the WOB) was more efficient than what was found by the GWO technique.

As mentioned, the BBO-ANN is the most accurate model of this study, but for answering the question “Which one of the neural-based ensembles is more efficient?” the complexity and time-effectiveness should be considered as well. It was shown that the BBO needs more time (about 2.9 times) and a larger population (3 times) compared to GWO to achieve the best solution. Notably, the difference between the learning error (i.e., the MSE) was approximately 2 units. Here, when time or computational ease comes out as determinant factors, utilizing the GWO is a better choice for optimizing the ANN, especially when it has provided satisfying accuracy. However, when the accuracy of the prediction is a more crucial factor, employing the BBO seems more reasonable.

### Investigating the effect of input variable dimension

Pearson’s method has previously been applied to sort the strength of WOB dependency on the depth, RPM, ROP, and GPM. This section investigates the effect of different combinations of input variables on both the accuracy and training time of the BBO-ANN model. As Table 3 shows, the BBO-ANN accuracy toward WOB predictions decreases by decreasing the input variable dimension from four (Depth, RPM, ROP, GPM) to two (Depth, RPM). It can also be seen that decreasing the input variable dimension has a slight impact on the BBO-ANN training time. Indeed, the training stage of the BBO-ANN model with four, three, and two input variables takes 31.17, 30.72, and 30.42 min, respectively.

**Table 3:** Checking the effect of input variable dimension on the BBO-ANN model performance.

Input variables	MAE		MSE		Training time* (min)
	Training	Testing	Training	Testing	
Depth, RPM, ROP, GPM	2.70	2.75	12.73	14.06	31.17
Depth, RPM, ROP	3.01	3.25	16.36	17.86	30.72
Depth, RPM	3.64	3.57	22.07	20.82	30.42

* Intel(R) Pentium(R) CPU G4400 @ 3.30 GHz, RAM 4.00 GB.

### Comparison with other techniques

This section compares the BBO-ANN accuracy toward the WOB prediction with MLR^37^, SVR^38^, ANFIS^39^, and GMDH^40^. Equation (14) introduces the linear correlation between WOB and all four input variables.

14 $${\text{WOB }} = \, - {16}.0{7 } + \, 0.00{748 } \times {\text{ Depth }} + \, 0.0{262 } \times {\text{ RPM }} - \, 0.{1217 } \times {\text{ ROP }} + \, 0.0{126 } \times {\text{ GPM}}$$

Table 4 relies on the presented MAEs and MSEs by the BBO-ANN, MLR, SVR, ANFIS, and GMDH in the training and testing steps to compare the models’ accuracy. This comparison analysis clarifies that the BBO-ANN accuracy in both the training and testing stages is better than all the other checked techniques. After the BBO-ANN, the adaptive neuro-fuzzy inference system with a cluster radius of 0.5 is the best intelligent tool to predict WOB from Depth, RPM, ROP, and GPM. This ANFIS model estimates the training dataset with MAE = 2.96 and MSE = 15.51. It also predicts the testing group with MAE = 2.95 and MSE = 15.32. The support vector regression with the linear kernel is also the worst model among the tested tools to estimate the WOB. It has MAE = 3.65 and MSE = 21.48 in the training step and MAE = 3.60 and MSE = 21.42 in the testing step.

**Table 4:** Comparing the BBO-ANN reliability for the WOB prediction with other techniques.

Model	MAE		MSE	
	Training	Testing	Training	Testing
BBO-ANN	2.70	2.75	12.73	14.06
ANFIS with cluster radius of 0.5	2.96	2.95	15.51	15.32
GMDH	3.11	3.13	16.83	17.36
MLR	3.62	3.77	21.16	22.56
SVR with linear kernel	3.65	3.60	21.48	21.42

### Extracting the neural predictive formula

Lastly, due to the superiority of the BBO-based ensemble, the governing formula of this network, composed of the ANN optimized parameters, is extracted and presented in Eq. (15). This equation addresses the mathematical rule established in the last layer of the used MLP (i.e., the output layer). Also, the optimized weights and biases of the middle layer (i.e., hidden layer) are presented in Table 5.

15 $$\begin{aligned} {\text{WOB}} & { = }1.3784 \times {\text{Z}}_{1} + 2.4171 \times {\text{Z}}_{2} + 0.99494 \times {\text{Z}}_{3} \\ & \quad - 1.3479 \times {\text{Z}}_{4} - 2.0371 \times {\text{Z}}_{5} \\ & \quad + 3.0183 \times {\text{Z}}_{6} - 4.1156 \times {\text{Z}}_{7} \\ & \quad - 9.0499 \times {\text{Z}}_{8} - 1.4611 \times {\text{Z}}_{9} + 15.6774 \\ \end{aligned}$$

where Z_1_, Z_2_, …, and Z_9_ are calculated as follows:

**Table 5:** Optimized weight and biases of the BBO-ANN model.

Neurons	Z_i_ = tansig (W_i1_ × Depth + W_i2_ × RPM + W_i3_ × ROP + W_i4_ × GPM + b_i_)				
	W_i1_	W_i2_	W_i3_	W_i4_	b_i_
1	− 0.17736	2.9165	− 10.6344	1.132	− 7.2108
2	0.061305	− 0.11876	1.0075	− 0.2992	− 20.8015
3	0.048444	0.43394	− 16.0667	− 0.34092	− 8.79
4	0.016888	− 0.41251	− 7.0085	0.12611	− 14.5611
5	− 0.01797	0.14339	0.1447	0.020829	18.7197
6	0.007208	0.063797	− 0.20392	− 0.00888	− 21.1413
7	0.008477	0.067772	− 0.0428	− 0.1278	22.1783
8	0.000077	− 0.00101	0.096582	− 0.00015	0.081523
9	0.001754	0.089777	− 0.71713	− 0.0177	3.0599

### Model interpretability

The drilling industry will greatly benefit from applying this novel neural-evolutionary framework for determining WOB in drilling operations. In the upstream oilfield industry, where cost-effective drilling operations are vital, an accurate calculation of WOB is essential for improving drilling processes. The model could represent the complex interactions affecting WOB by including drilling depth, drill string rotational speed, rate of penetration, and volumetric flow rate.

With the use of this knowledge, drilling program decisions may be made with great consideration, resulting in parameters that considerably impact the rate of penetration into the formation. Its adaptability results from the framework's success in predicting WOB under various conditions, including those where mud qualities, drill bit types, and hydraulics are taken into account. Using such sophisticated prediction models can result in better-informed and optimized drilling procedures, which will eventually help the drilling industry succeed as it seeks out cost- and efficiency-saving opportunities.

The application of our study holds tremendous potential for operational companies looking for cutting-edge solutions in the complex and expensive world of drilling operations. Automated drilling rigs, often known as "smart" drilling rigs, are one example. These rigs use cutting-edge technology to automate several drilling operations, such as wellbore navigation and directional drilling^41^. These systems frequently use real-time data analysis and machine learning to improve drilling circumstances and make decisions. Real-time data analytics tools are also being integrated more and more frequently. These platforms analyze data from various sensors and drilling equipment to deliver quick insights into drilling performance, empowering operators to make decisions based on data immediately^42^.

## Conclusions

The main objective of this study was to investigate the capability of the evolutionary optimization algorithm to accomplish the training process of artificial neural networks to predict the WOB as a function of different combinations of depth, RPM, ROP, and GPM. To this end, grey wolf and biogeography-based optimization algorithms were coupled with the ANN to adjust weights and biases. Based on the executed sensitivity analysis, the BBO-ANN model needed a larger population size and more computational time than the GWO-ANN. The obtained error criteria of MSE and MAE revealed that both applied algorithms perform satisfactorily in enhancing the learning capability of the ANN. Moreover, the BBO surpassed GWO in prevailing the computational drawbacks of the ANN, and also, the BBO-ANN produced the most consistent results, followed by GWO-ANN and LM-ANN. The effect of input variable dimension on the accuracy and training time showed that the BBO-ANN provided better WOB prediction when it was constructed based on all four input variables instead of utilizing those with the highest linear correlation (i.e., depth and RPM or depth, RPM, and ROP). In addition, the constructed BBO-ANN predicted the actual WOB data with better accuracy than the MLR, ANFIS, SVR, and GMDH. The BBO-ANN formula was presented to be used for directly estimating the WOB, influenced by four effective factors, including drilling depth, drill string rotational speed, rate of penetration, and volumetric flow rate.

## Data Availability

The utilized data in this study is available upon reasonable request from the corresponding author.

## References

[1] Koroteev D, Tekic Z (2021). Artificial intelligence in oil and gas upstream: Trends, challenges, and scenarios for the future. Energy AI.

[2] Palmer, A. *Introduction to Petroleum Exploration and Engineering* (World Scientific, 2017).

[3] Ma T, Chen P, Zhao J (2016). Overview on vertical and directional drilling technologies for the exploration and exploitation of deep petroleum resources. Geomech. Geophys. Geo-Energy Geo-Resources.

[4] Wang Y, Lou M, Wang Y, Fan C, Tian C, Qi X (2023). Experimental investigation of the effect of rotation rate and current speed on the dynamic response of riserless rotating drill string. Ocean Eng..

[5] Tunio SQ, Tunio AH, Ghirano NA, Irawan S (2011). Is it possible to ignore problems rising during vertical Drilling? A review. Res. J. Appl. Sci. Eng. Technol..

[6] Galle EM, Woods HB (1963). Best constant weight and rotary speed for rotary rock bits. Drill. Prod. Pract..

[7] Alum, M. A. & Egbon, F. Semi-analytical models on the effect of drilling fluid properties on rate of penetration (ROP). In *Nigeria Annual International Conference and Exhibition* (Society of Petroleum Engineers, 2011).

[8] Moraveji MK, Naderi M (2016). Drilling rate of penetration prediction and optimization using response surface methodology and bat algorithm. J. Nat. Gas Sci. Eng..

[9] Motahhari, H. R., Hareland, G., Nygaard, R. & Bond, B. Method of optimizing motor and bit performance for maximum ROP. In *Canadian International Petroleum Conference* (Petroleum Society of Canada, 2007).

[10] Fear MJ (1999). How to improve rate of penetration in field operations. SPE Drill. Complet..

[11] Xu H, Wu X, Khandakar A (2022). Estimation of the methanol loss in the gas hydrate prevention unit using the artificial neural networks: Investigating the effect of training algorithm on the model accuracy. Energy Sci. Eng..

[12] Khosravanian R, Sabah M, Wood DA, Shahryari A (2016). Weight on drill bit prediction models: Sugeno-type and Mamdani-type fuzzy inference systems compared. J. Nat. Gas Sci. Eng..

[13] Yin H, Zhang G, Wu Q, Yin S, Soltanian MR, Thanh HV, Dai Z (2023). A deep learning-based data-driven approach for predicting mining water inrush from coal seam floor using micro-seismic monitoring data. IEEE Trans. Geosci. Rem. Sens..

[14] Yin H, Wu Q, Yin S, Dong S, Dai Z, Soltanian MR (2023). Predicting mine water inrush accidents based on water level anomalies of borehole groups using long short-term memory and isolation forest. J. Hydrol..

[15] Zhao Y, Noorbakhsh A, Koopialipoor M, Azizi A, Tahir MM (2019). A new methodology for optimization and prediction of rate of penetration during drilling operations. Eng. Comput..

[16] Tie Y, Rui X, Shi-Hui S, Zhao-Kai H, Jin-Yu F (2023). A real-time intelligent lithology identification method based on a dynamic felling strategy weighted random forest algorithm. Pet. Sci..

[17] Fathi M, Parian JA (2021). Intelligent MPPT for photovoltaic panels using a novel fuzzy logic and artificial neural networks based on evolutionary algorithms. Energy Rep..

[18] Anemangely M, Ramezanzadeh A, Tokhmechi B, Molaghab A, Mohammadian A (2018). Drilling rate prediction from petrophysical logs and mud logging data using an optimized multilayer perceptron neural network. J. Geophys. Eng..

[19] Wang J (2022). Estimating the relative crystallinity of biodegradable polylactic acid and polyglycolide polymer composites by machine learning methodologies. Polymers (Basel)..

[20] Schmidt J-S, Osebold R (2017). Environmental management systems as a driver for sustainability: State of implementation, benefits and barriers in German construction companies. J. Civ. Eng. Manag..

[21] Alibak AH (2022). Developing a hybrid neuro-fuzzy method to predict carbon dioxide (CO_2_) permeability in mixed matrix membranes containing SAPO-34 zeolite. Membranes (Basel)..

[22] Sohani A, Dehbashi M, Delfani F, Hoseinzadeh S (2023). Optimal techno-economic and thermo-electrical design for a phase change material enhanced renewable energy driven polygeneration unit using a machine learning assisted lattice Boltzmann method. Eng. Anal. Bound. Elem..

[23] McCulloch WS, Pitts W (1943). A logical calculus of the ideas immanent in nervous activity. Bull. Math. Biophys..

[24] Shi Y, Song X, Song G (2021). Productivity prediction of a multilateral-well geothermal system based on a long short-term memory and multi-layer perceptron combinational neural network. Appl. Energy.

[25] Moré, J. J. The Levenberg-Marquardt algorithm: Implementation and theory. In *Numerical Analysis* 105–116 (Springer, 1978).

[26] Abdollahzadeh M (2022). Estimating the density of deep eutectic solvents applying supervised machine learning techniques. Sci. Rep..

[27] Altan A, Karasu S, Zio E (2021). A new hybrid model for wind speed forecasting combining long short-term memory neural network, decomposition methods and grey wolf optimizer. Appl. Soft Comput..

[28] Mirjalili S, Mirjalili SM, Lewis A (2014). Grey wolf optimizer. Adv. Eng. Softw..

[29] Dehghani M (2019). Prediction of hydropower generation using grey wolf optimization adaptive neuro-fuzzy inference system. Energies.

[30] Bozorg-Haddad, O. *Advanced optimization by nature-inspired algorithms*. (Springer, 2018).

[31] Muro C, Escobedo R, Spector L, Coppinger RP (2011). Wolf-pack (Canis lupus) hunting strategies emerge from simple rules in computational simulations. Behav. Process..

[32] Simon D (2008). Biogeography-based optimization. IEEE Trans. Evol. Comput..

[33] Mirjalili S, Mirjalili SM, Lewis A (2014). Let a biogeography-based optimizer train your multi-layer perceptron. Inf. Sci. (NY).

[34] Bhattacharya A, Chattopadhyay PK (2010). Solving complex economic load dispatch problems using biogeography-based optimization. Expert Syst. Appl..

[35] Roy PK, Ghoshal SP, Thakur SS (2010). Biogeography based optimization for multi-constraint optimal power flow with emission and non-smooth cost function. Expert Syst. Appl..

[36] Hadidi A (2015). A robust approach for optimal design of plate fin heat exchangers using biogeography based optimization (BBO) algorithm. Appl. Energy.

[37] Abdollahi SA, Ranjbar SF (2023). Modeling the CO2 separation capability of poly (4-methyl-1-pentane) membrane modified with different nanoparticles by artificial neural networks. Sci. Rep..

[38] Shi C, Pei W, Jin C, Alizadeh A, Ghanbari A (2023). Prediction of the SnO2-based sensor response for hydrogen detection by artificial intelligence techniques. Int. J. Hydrog. Energy.

[39] Bagherzadeh A (2022). Developing a global approach for determining the molar heat capacity of deep eutectic solvents. Meas. J. Int. Meas. Confed..

[40] Abdollahi SA, Ranjbar SF, Razeghi Jahromi D (2023). Applying feature selection and machine learning techniques to estimate the biomass higher heating value. Sci. Rep..

[41] Abdulmalek, A. S. *et al.* Prediction of rate of penetration of deep and tight formation using support vector machine. in *SPE Kingdom of Saudi Arabia Annual Technical Symposium and Exhibition* (OnePetro, 2018).

[42] Al-AbdulJabbar A (2020). Prediction of the rate of penetration while drilling horizontal carbonate reservoirs using the self-adaptive artificial neural networks technique. Sustainability.

